# Molecular epidemiology of hereditary ataxia in Finland

**DOI:** 10.1186/s12883-021-02409-z

**Published:** 2021-10-02

**Authors:** Joonas Lipponen, Seppo Helisalmi, Joose Raivo, Ari Siitonen, Hiroshi Doi, Harri Rusanen, Maria Lehtilahti, Mervi Ryytty, Markku Laakso, Fumiaki Tanaka, Kari Majamaa, Laura Kytövuori

**Affiliations:** 1 Research Unit of Clinical Neuroscience, Medical Research Center Oulu, Oulu University Hospital and University of Oulu, P.O. Box 5000, 90014 Oulu, Finland; 2 Department of Neurology, Oulu University Hospital, Oulu, Finland; 3 Institute of Clinical Medicine, Internal Medicine, University of Eastern Finland, Kuopio, Finland; 4 Department of Neurology and Stroke Medicine, Yokohama City University Graduate School of Medicine, Yokohama, Japan

**Keywords:** CANVAS, Hereditary ataxia, Molecular epidemiology, Repeat expansion

## Abstract

**Background:**

The genetics of cerebellar ataxia is complex. Hundreds of causative genes have been identified, but only a few cause more than single cases. The spectrum of ataxia-causing genes differs considerably between populations. The aim of the study was to investigate the molecular epidemiology of ataxia in the Finnish population.

**Patients and methods:**

All patients in hospital database were reviewed for the diagnosis of unspecified ataxia. Acquired ataxias and nongenetic ataxias such as those related to infection, trauma or stroke were excluded. Sixty patients with sporadic ataxia with unknown etiology and 36 patients with familial ataxia of unknown etiology were recruited in the study. Repeat expansions in the SCA genes (*ATXN1*, *2*, *3*, *7*, *8/OS*, *CACNA1A, TBP*), *FXN*, and *RFC1* were determined. Point mutations in *POLG*, *SPG7* and in mitochondrial DNA (mtDNA) were investigated. In addition, DNA from 8 patients was exome sequenced.

**Results:**

A genetic cause of ataxia was found in 33 patients (34.4%). Seven patients had a dominantly inherited repeat expansion in *ATXN8/OS*. Ten patients had mitochondrial ataxia resulting from mutations in nuclear mitochondrial genes *POLG* or *RARS2*, or from a point mutation m.8561C > G or a single deletion in mtDNA. Interestingly, five patients were biallelic for the recently identified pathogenic repeat expansion in *RFC1*. All the five patients presented with the phenotype of cerebellar ataxia, neuropathy, and vestibular areflexia (CANVAS). Moreover, screening of 54 patients with Charcot-Marie-Tooth neuropathy revealed four additional patients with biallelic repeat expansion in *RFC1*, but none of them had cerebellar symptoms.

**Conclusions:**

Expansion in *ATXN8/OS* results in the majority of dominant ataxias in Finland, while mutations in *RFC1* and *POLG* are the most common cause of recessive ataxias. Our results suggest that analysis of *RFC1* should be included in the routine diagnostics of idiopathic ataxia and Charcot-Marie-Tooth polyneuropathy.

**Supplementary Information:**

The online version contains supplementary material available at 10.1186/s12883-021-02409-z.

## Background

About 80% of the ataxias are sporadic, in which alcohol-related ataxia and non-hereditary degenerative ataxias are the most common [1]. The majority of the hereditary ataxias are caused by dominantly inherited trinucleotide repeat expansion mainly in *ATXN*s, and these ataxias form a subgroup of spinocerebellar ataxias (SCAs). In addition, ataxia is a common feature in the mitochondrial disorders caused by a defect in mitochondrial DNA or in nuclear genome.

Recessively inherited Friedreich’s ataxia (FRDA) is the most frequent heritable ataxia in Southern and Western Europe, but it seems to be rather rare in Northern Europe [1–3]. Among the autosomal dominant ataxias SCA2 and SCA3 are the most prevalent among Caucasians [4, 5], whereas in the Finnish population SCA2 is very rare and SCA3 is completely absent [6]. By contrast, *ATXN8/OS*-related SCA8 and mitochondrial recessive ataxia syndrome (MIRAS) resulting from mutations in *POLG* have been reported in Finland [6, 7]. The differences may be explained by population history, as small founder population and rapid expansion associated with geographic isolation of subpopulations have resulted in unique genetic make-up and Finnish disease heritage [8].

New sequencing techniques have enabled identification of novel ataxia-causing mutations but, unfortunately, there are almost 600 genes associated with ataxic disorders and a single gene resolves only few cases. In addition, exome sequencing does not cover intronic or intergenic regions, where disease-associated mutations may locate as well. Indeed, the most recent ataxia-causing mutation has been found in the intronic region of *RFC1* gene [9, 10].

We ascertained here a population-based cohort of patiens with ataxia of unknown etiology from a defined population in northern Finland and investigated the molecular etiology of their ataxia. We characterized the genetic spectrum of ataxia and found that the pentanucleotide repeat expansion in the *RFC1* gene is a common cause of ataxia in Finland.

## Subjects and methods

Oulu University Hospital (OUH) is the sole provider of specialized neurological services in the province of Northern Ostrobothnia with a population of 412,830 on 31 December 2019. Adult patients who had visited OUH between 1 November 1997 – 31 October 2019 and had a diagnosis of unspecified hereditary ataxia or unspecified ataxia (ICD-10: G11.9, R27.0) were identified from the OUH patient database. The search yielded 408 patients and after exclusions (see Additional file 1), a pre-visit questionnaire was mailed to 125 patients. Eighty-one patients (64.8%) responded and five additional patients were identified during the study at the outpatient clinic. Furthermore, 11 patients were included from the cohort of 26 patients identified in a similar study covering the years 1976–1994 [11]. One patient withdrew consent leaving a total of 96 patients. Controls for *RFC1* haplotyping consisted of 269 anonymous, healthy blood donors from Finnish Red Cross.

A separate cohort of polyneuropathy consisted of 66 patients with Charcot-Marie-Tooth (CMT) disease type 2, 19 patients with CMT1 and 14 patients with intermediate type CMT [12]. The screening of *RFC1* was carried out in the 54 patients with CMT, who had not received a genetic diagnosis in the previous study.

### Molecular genetics

DNA was extracted from the blood samples using QIAsymphony DSP DNA kit with QIAsymphony robot and QIAamp DNA Blood Kit (QIAGEN, Hilden, Germany). A flowchart describing genetic investigation is shown in Additional file 1. Briefly, a fluorescent PCR and fragment analysis was used to genotype SCA1 (*ATXN1*), SCA2 (*ATXN2*), SCA3 (*ATXN3*), SCA6 (*CACNA1A*), SCA7 (*ATXN7*), SCA8 (*ATXN8OS*), SCA17 (*TBP*) and *FXN*. Fragment analyses were done using a GeneScan™ LIZ500 size standard with ABI 3500xL Genetic Analyzer (Thermo Fisher Scientific, Waltham, MA, U.S.A.). The size of the fragments was determined using GeneMapper® 5.0 software (Thermo Fisher Scientific).

**Fig. 1:**
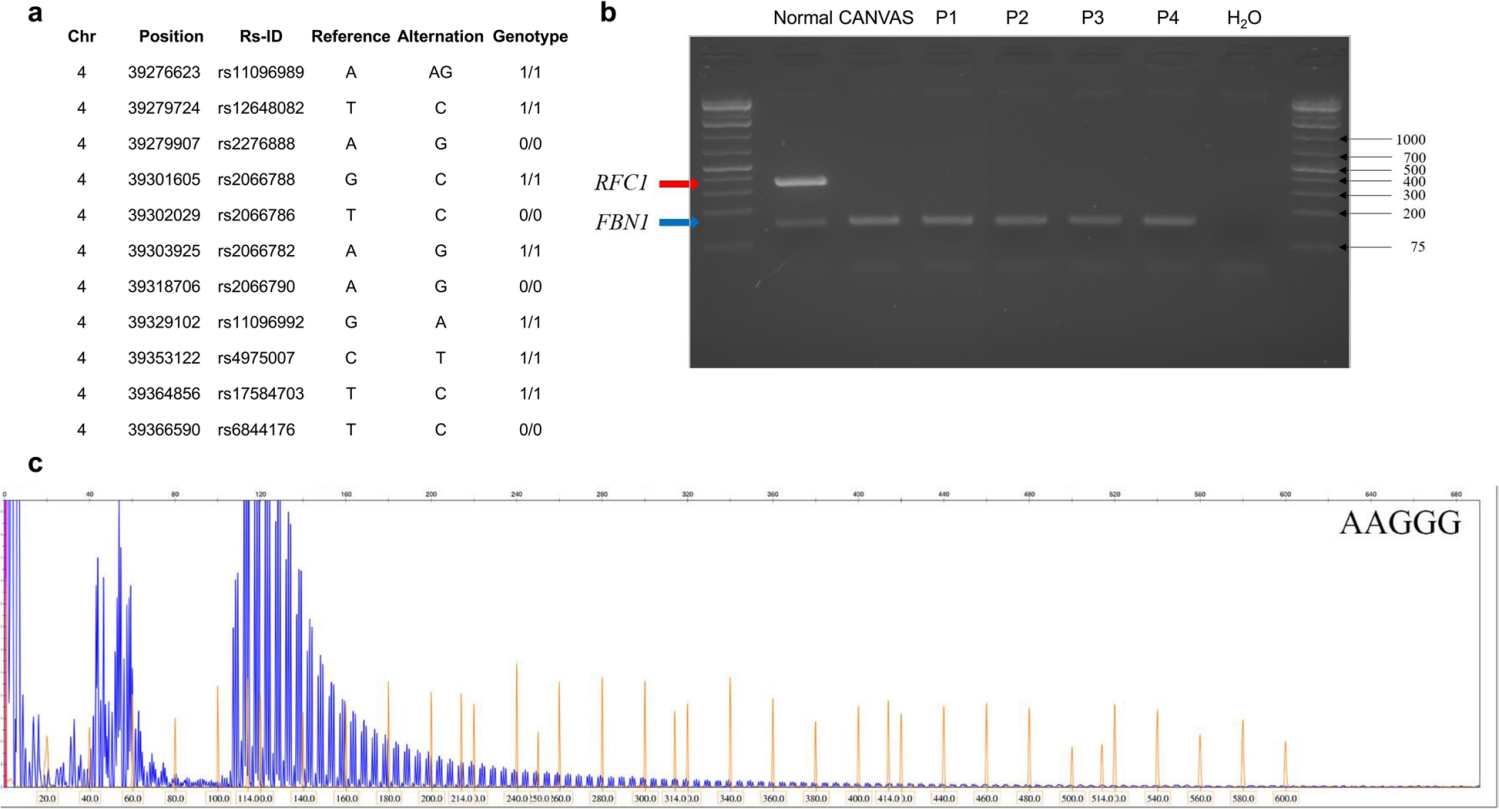
*RFC1* in Finnish patients with ataxia. **A** Core haplotype around the pathogenic expansion in the *RFC1* gene. **B** Multiplex PCR of *RFC1* normal allele and *FBN1* as control in four patients with biallelic expansion. CANVAS, positive control; P, patient; H_2_O, negative control. **C** Example of repeat-primed PCR in one patient with biallelic AAGGG expansion

Common mutations in *POLG*, the p.Ala510Val mutation in *SPG7*, the m.3243A > G and m.8344A > G mutations in mtDNA were examined and *MT-ATP6* and *MT-ATP8* genes were sequenced. The variants rs6844176, rs17584703, rs11096992 and rs2066790 defining the *RFC1* haplotypes were investigated using restriction fragment length polymorphism with FastDigest® *RseI*, *TaaI*, *BseJI* and *Eco105I* (Thermo Fisher Scientific), respectively. Furthermore, the haplotypes were investigated in 269 healthy controls. Exome sequencing data were used to create a detailed haplotype associated with the pathogenic expansion (Fig. 1).

XL-PCR of *RFC1* expansion was carried as previously [9]. *PhireII*™ Hot Start Polymerase was used to confirm XL-PCR results with the same primers by using protocols for short and long fragments. Flanking multiplex PCR for *FBN1* as a control and *RFC1* was done using TaKaRa Ex Taq Hot Start® polymerase (Fig. 1). Fluorescent-labeled repeat-primed PCR-products (RP-PCR) were analyzed with a GeneScan™ 600LIZ standard (Thermo Fisher Scientific). All PCR primers and reaction conditions are available in Additional info.

DNA library preparation for exome sequencing was carried out using Nextera Rapid Capture Exome Library kit, according to the manufacturer’s protocol (Illumina, San Diego, CA, U.S.A.) in Biocenter Oulu Sequencing Center. The sequencing was done using Illumina NextSeq550 platform. Data pre-processing and quality control has been described previously [13]. The Q30 value of the contigs was an average of approximately 92.85%. Variants were filtered by minor allele frequency less than 0.1%. Variants in 562 ataxia-causing genes were investigated in eight patients that were exome sequenced (see Additional file 1).

## Results

Ninety-six patients (48 men) from 89 families participated in the study. Median age of onset was 40 years (range, 1–75 years) and median disease duration was 19 years (range, 1–68 years). A detailed family history was obtained for 89 patients, including 27 patients with ataxia in first-degree relatives.

Twenty-two patients had a previous genetic diagnosis and 11 patients received a new diagnosis (Table 1). Clinical findings of the 33 patients with a confirmed genetic diagnosis are shown in Table 2. Expansion in *ATXN8/OS* (SCA8) was the most common cause of dominantly inherited ataxia, while the homozygous p.Trp748Ser mutation in *POLG* and the biallelic expansion in *RFC1* were the most common causes of recessively inherited ataxia. Three patients had autosomal recessive spastic ataxia of Charlevoix-Saguenay (ARSACS) and three patients had point mutations in *CACNA1A* [14, 15]. Two siblings had a mitochondrial ataxia syndrome resulting from m.8561C > G mutation in the overlapping region of *MT-ATP6* and *MT-ATP8* [16]. Exome sequencing revealed a p.His605fs frameshift mutation in *ATM* with a previously reported pathogenic mutation p.Gly2891Asp in one patient [17]. Other rare causes included a single deletion in mtDNA and mutations in *SAMD9L*, *RARS2* and *FRMI*, respectively.

**Table 1:** Genetic causes of ataxia in Finnish patients with ataxic disorders

Gene	Inheritance	Mutations (Patient ID and repeat number when available)	Patients (N)
*ATXN8/OS*	AD	trinucleotide repeat expansion (P1 118, P2 98, P3 139, P4 190, P5 90, P6 125, P7 90)	7
*CACNA1A*	AD	c.3414dup p.Lys1139fs (P22), c.5629-2A > G^15^ (P23, P24)	3
*SAMD9L*	AD	c.2672 T > C p.Ile891Thr	1
*FMRI*	XLD	trinucleotide repeat expansion (P31 60)	1
*POLG*	AR	c.2243C > G p.Trp748Ser/ c.2243C > G p.Trp748Ser	6
*RFC1*	AR	pentanucleotide repeat expansion AAGGG_exp_	5
*SACS*	AR	[c.3298G > A p.Glu1100Lys; c.4466A > G, p.Asn1489Ser]/c.4076 T > C p.Met1359Thr^14^	3
*FXN*	AR	trinucleotide repeat expansion (P25 970/970)	2
*RARS2*	AR	c.773G > A p.Arg258His/p.Ala369fs	1
*ATM*	AR	c.1813del p.His605fs/c.8672G > A p.Gly2891Asp	1
*MT-ATP6/8*	M	m.8561C > G	2
mtDNA	de novo	mtDNA deletion 4.5 kb	1
No genetic diagnosis			63
Total			96

GenBank reference sequences: *ATM*: NM_001351834.2, NP_000042.3; *CACNA1A*: NM_001127221.2, NP_001120693.1; mtDNA: NC_012920; *POLG*: NM_002693.3, NP_001119603.1; *RARS2*: NM_020320.5, NP_001337434.1; *SACS*: NM_014363.6, NP_055178.3; *SAMD9L*: NM_001303496.3, NP_001290425.1

*AD* autosomal dominant, *AR* autosomal recessive, *M* mitochondrial, *XLD* X-chromosomal dominant

**Table 2:** Clinical findings in Finnish patients with genetically confirmed ataxic disorders

**ID**	**Sex**	**AOO**	**AAE**	**Gene**	**CI**	**DM**	**E**	**HI**	**VI**	**PNP**	**Tonus**	**EyeM**	**Dysph**	**Dysar**	**KPS **[18]
1	F	NA	NA	*ATXN8/OS*	-	-	-	-	-	-	NA	NA	NA	NA	NA
2	F	50	76	*ATXN8/OS*	-	-	-	+	-	-	N	N	+	-	80
3	F	57	72	*ATXN8/OS*	-	-	-	-	-	-	N	N	-	+	80
4	F	36	64	*ATXN8/OS*	-	-	-	-	-	-	N	N	+	+	70
5	F	35	36	*ATXN8/OS*	-	-	-	-	-	-	N	I	-	+	80
6	F	33	36	*ATXN8/OS*	-	-	-	-	-	-	N	N	+	+	80
7	M	40	64	*ATXN8/OS*	+	+	-	-	+	+	NA	NA	+	+	80
8	F	32	55	*POLG*	-	-	+	-	-	+	N	I	-	+	20
9	M	45	59	*POLG*	+	-	-	+	-	+	N	N	-	-	70
10	M	24	45	*POLG*	-	-	+	-	+	+	N	I	+	+	60
11	M	50	58	*POLG*	-	-	-	-	-	-	N	NA	-	+	80
12	F	30	43	*POLG*	-	-	-	-	-	+	N	I	-	-	80
13	F	40	42	*POLG*	+	+	-	-	+	+	N	I	+	+	80
14	F	40	72	*RFC1*	+	-	+	-	-	+	N	I	+	+	40
15	M	45	57	*RFC1*	-	-	-	-	-	+	N	I	-	+	60
16	F	52	64	*RFC1*	-	-	-	-	+	+	N	I	-	+	70
17	F	64	74	*RFC1*	-	+	-	-	-	+	N	I	+	+	50
18	M	48	71	*RFC1*	-	-	-	-	-	+	N	I	-	-	90
19	F	20	58	*SACS*	-	-	-	-	-	+	S, R	I	-	+	50
20	M	10	65	*SACS*	-	-	-	-	-	+	S	I	-	+	40
21	M	7	63	*SACS*	-	-	-	-	-	+	S	I	-	+	40
22	M	1	NA	*CACNA1A*	-	-	-	-	+	-	N	I	-	+	NA
23	M	10	44	*CACNA1A*	-	-	-	-	-	-	N	N	-	-	90
24	F	11	20	*CACNA1A*	-	-	-	-	-	-	N	N	-	-	90
25	F	5	37	*FXN*	-	+	-	-	-	+	S	I	-	+	50
26	F	22	29	*FXN*	-	-	-	-	-	+	S	I	-	-	80
27	M	20	64	*MT-ATP6/8*	-	+	-	+	-	+	N	N	+	+	60
28	F	20	59	*MT-ATP6/8*	-	+	-	-	-	-	N	N	-	+	60
29	F	32	38	*SAMD9L*	+	-	-	-	-	-	N	I	+	-	80
30	M	70	85	*ATM*	+	-	-	-	-	-	N	I	+	+	50
31	M	57	63	*FMRI*	+	-	-	-	-	-	N	N	+	+	80
32	F	2	28	*RARS2*	+	-	+	-	-	-	N	I	+	-	70
33	M	4	26	mtDNA del	+	+	-	+	+	-	N	I	-	-	40

*AAE* age at examination, *AOO* age of onset, *CI* cognitive impairment, *del* deletion, *DM* diabetes mellitus, *Dysar* dysarthria, *dysph* dysphagia, *E* epilepsy, *EyeM* eye movements, *HI* hearing impairment, *I* impaired eye movements include double vision, impaired saccades, impaired smooth pursuit or nystagmus, *KPS* Karnofsky performance score, *N* normal, *NA* not analyzed, *PNP* polyneuropathy, *R* rigidity, *S* spasticity, *VI* visual impairment

Axonal polyneuropathy is a core feature of CANVAS and, hence, we investigated *RFC1* expansion in a separate cohort of 54 patients with a clinical diagnosis of Charcot-Marie-Tooth disease [12]. Four patients with biallelic repeat expansion were found, but none of them presented with cerebellar symptoms.

### RFC1 haplotypes

The five patients with the pathogenic AAGGG expansion (AAGGG)_exp_ shared a homozygous haplotype defined by four single-nucleotide polymorphisms. The frequency of this haplotype was 12.8% among the patients with ataxia and 10.4% among the controls. Interestingly, two controls with the homozygous haplotype harbored a single copy of a short pathogenic expansion. A detailed haplotype created from exome sequencing data of the *RFC1* patients was identical with those reported previously (Fig. 1) [10, 19].

## Discussion

We found that the *ATXN8/OS* trinucleotide repeat expansion is the most prevalent cause of dominantly inherited ataxia in Finland confirming a previous observation [6]. Common recessive causes were p.Trp748Ser in *POLG* and the recently discovered intronic expansion in *RFC1*. SCA3, FRDA and *SPG7*-related ataxia have been reported to be the most common hereditary ataxias among Europeans [4, 20], but we found only two patients with FRDA and none with SCA3 or mutations in *SPG7*. Altogether, 34% of our patients received a genetic diagnosis and the three most common genetic causes accounted for half of the diagnoses. These frequencies are specific to Finns and emphasize genetic differences between Caucasian populations.

CANVAS is a new member in the syndromes that are caused by an intronic mutation [9, 10]. The biallelic expansion (AAGGG)_exp_ in *RFC1* was found in five patients that presented with ataxia, polyneuropathy and nystagmus resembling typical CANVAS [21]. Vestibular dysfunction is a core feature in CANVAS and, consistently, our patients had abnormal eye movements, but vestibular areflexia had been confirmed with caloric test only in one patient. Another patient had a more severe phenotype including epilepsy and cognitive impairment. Neither epilepsy nor cognitive impairment are features in typical CANVAS, but atypical phenotypes, such as dementia with Lewy bodies, have been reported recently [19]. The repeat expansion in *RFC1* is a new genetic cause of disease and it is probable that new features of the phenotype will be discovered. In addition, we found four patients with biallelic repeat expansion in *RFC1* among patients with Charcot-Marie-Tooth disease [12]. These numbers suggest that *RFC1* is among the three most common genetic causes of Charcot-Marie-Tooth disease in Finland together with *PMP22* duplication and p.His123Arg in *GDAP1*.

Two controls with the homozygous *RFC1* haplotype harbored a single copy of a short pathogenic expansion, but the risk of developing a large expansion is unknown for those who harbor a short form of the expansion. All subjects are potential carriers of the large expansion, if they harbor one or two copies of the haplotype that is associated with the large repeat expansion. However, the large expansion allele cannot be amplified in PCR and thus, heterozygous subjects may appear as homozygous for the normal-sized repeat allele even if they, in fact, are carriers of the large expansion. Segregation analyses with determination of the exact size of the expansion are required in order to estimate the risk of having an increase in the repeat number in the next generation.

A mitochondrial cause of ataxia was detected in ten patients including six patients with homozygous mutations in *POLG*, siblings with m.8561C > G [16], a patient with a single deletion in mtDNA and a patient with compound heterozygous mutations in *RARS2*. Recently, the m.8561C > T mutation has been reported in a patient with severe mitochondrial disorder [22] suggesting that *MT-ATP* genes may be mutational hotspots in the aetiology of ataxia. Mitochondrial defects have previously been found in > 20% of ataxia patients, especially in those with a syndromic phenotype [23]. Single patients were found to harbour mutations in *ATM*, *SAMD9L*, or *FMRI* that have occasionally been reported as a cause of inherited ataxia [24–26]. The mutation in *ATM* was found in exome sequencing, but the genetic cause remained unclear in the seven other patients that were analyzed for 562 ataxia-causing genes.

## Conclusions

We found that mutations in *ATXN8/OS*, *POLG* and *RFC1* are the most common genetic causes of ataxia in Finland. The pentanucleotide repeat expansion in *RFC1* is a frequent cause of polyneuropathy as well suggesting that routine diagnostic testing should be carried out in patients with suspected hereditary ataxia or polyneuropathy.

## Supplementary Information

**Additional file 1: Figure 1.** Selection criteria of the patients. **Figure 2.** Genetic investigations of the patients. **Table 1.** List of 562 genes analyzed from exome sequencing data.

**Additional file 2: Additional info.** Lab protocols.

## Data Availability

The phenotype-genotype data of the study are available in the manuscript. The lab protocols are available in the additional info of the manuscript. The mtDNA sequences are available in the GenBank with accession numbers MZ475290-MZ475297. Variants have been submitted to ClinVar (identifiers pending) and MitoMap (https://www.mitomap.org/MITOMAP). Due to privacy policies, the exome sequencing data cannot be publicly available.

## Abbreviations

CANVAS: Cerebellar ataxia, neuropathy, and vestibular areflexia
FRDA: Friedreich’s ataxia
MIRAS: Mitochondrial recessive ataxia syndrome
mtDNA: Mitochondrial DNA
OUH: Oulu University Hospital
RP-PCR: Repeat-primed polymerase chain reaction
SCA: Spinocerebellar ataxia
