# Supplementary material for: Molecular epidemiology of hereditary ataxia in Finland
Source: BMC Neurol. 2021 Oct 2;21:382. doi: 10.1186/s12883-021-02409-z (PMC8487109; doi:10.1186/s12883-021-02409-z)
Supplement: Supplementary file 1 — Additional file 1: Figure 1. Selection criteria of the patients. Figure 2. Genetic investigations of the patients. Table 1. List of 562 genes analyzed from exome sequencing data. [file 12883_2021_2409_MOESM1_ESM.pdf]

Molecular epidemiology of hereditary ataxia in Finland

Additional file

Joonas Lipponen[1,2], Seppo Helisalmi[3], Joose Raivo[3], Ari Siitonen[1,2], Hiroshi Doi[4], Harri Rusanen[1,2], Maria Lehtilahti[1,2], Mervi Ryytty[1,2], Markku Laakso[3], Fumiaki Tanaka[4], Kari Majamaa[1,2] and Laura Kytövuori[1,2]

[1]Research Unit of Clinical Neuroscience, Medical Research Center Oulu, Oulu University Hospital and University of Oulu, Oulu, Finland [2]Department of Neurology, Oulu University Hospital, Oulu, Finland, [3]Institute of Clinical Medicine, Internal Medicine, University of Eastern Finland, Kuopio, Finland, [4]Department of Neurology and Stroke Medicine, Yokohama City University Graduate School of Medicine, Yokohama, Japan

Additional figure 1. Selection criteria of the patients.

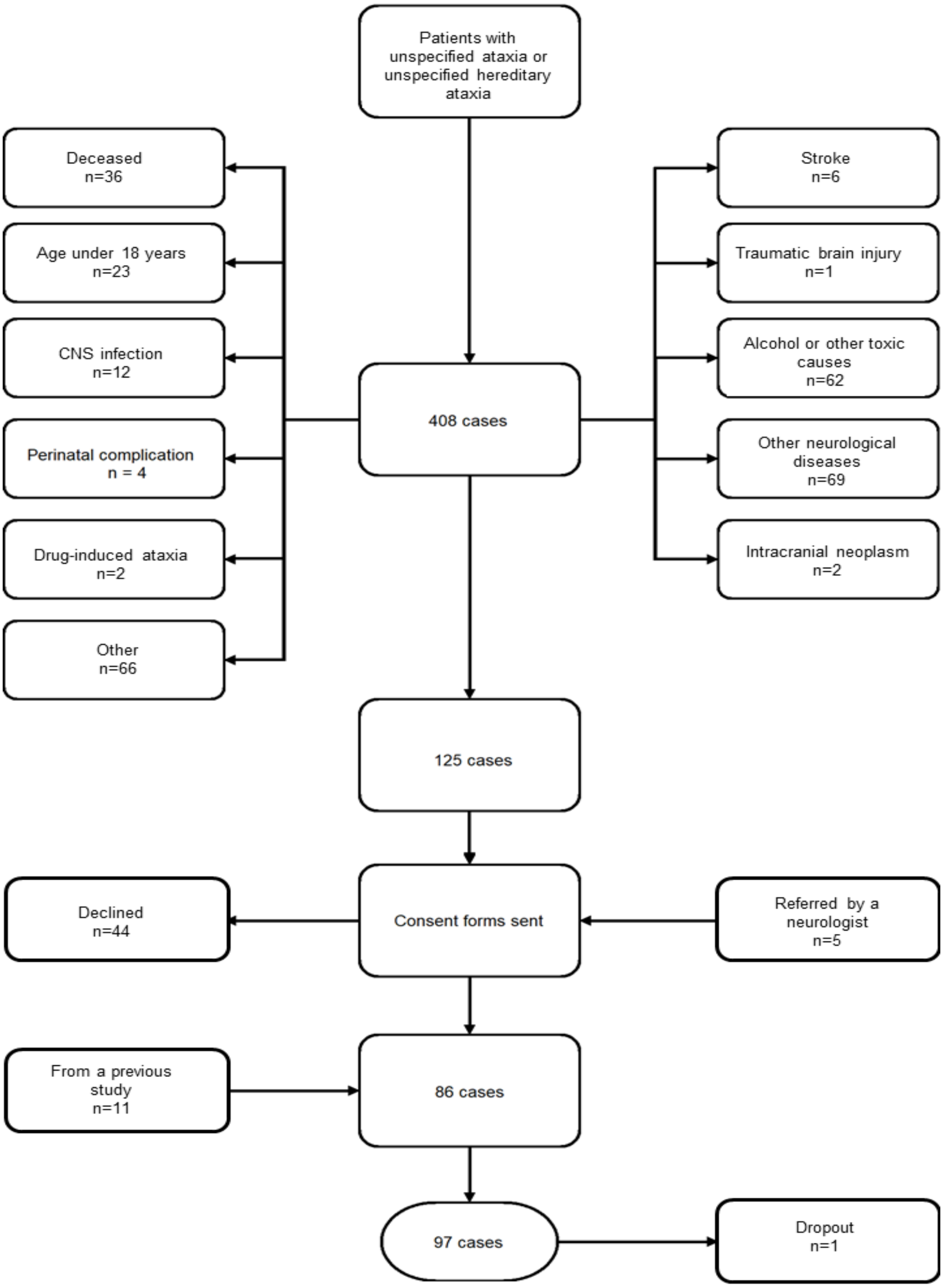

Additional figure 2. Genetic investigations of the patients.

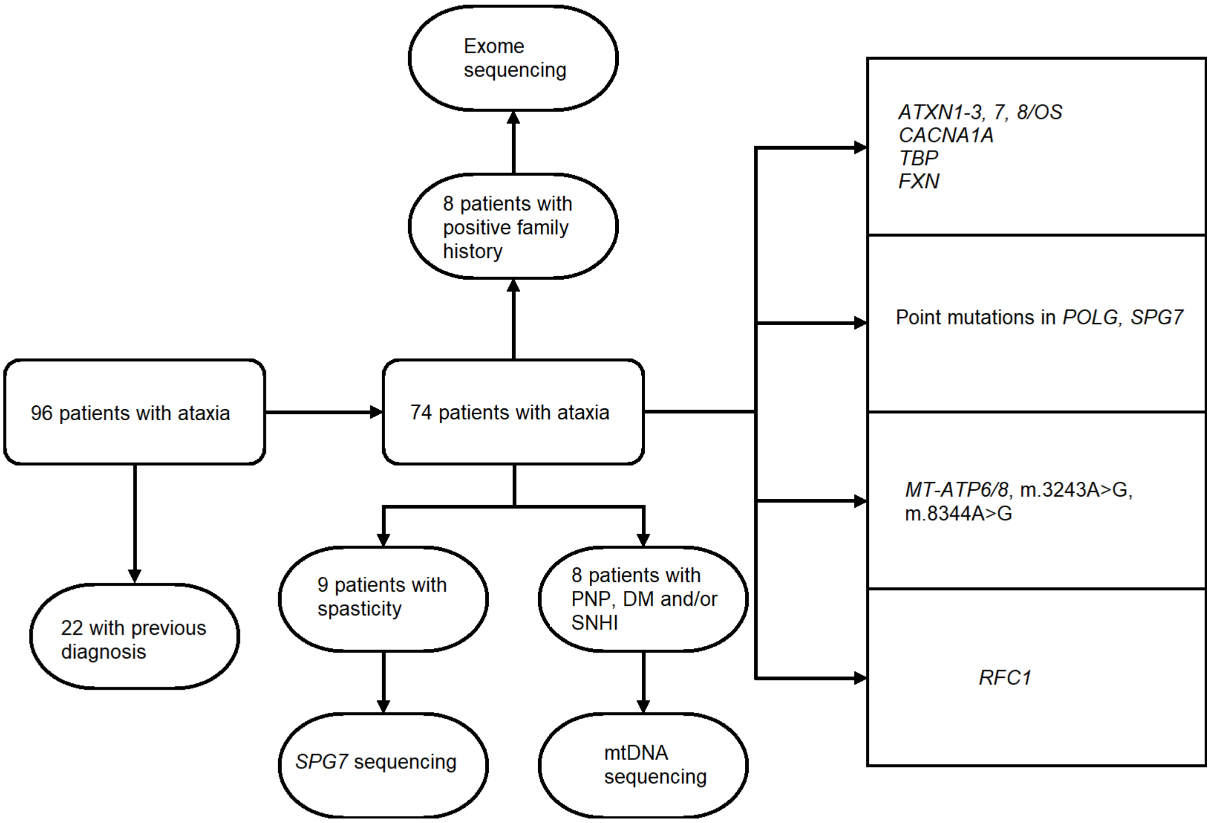

DM, diabetes mellitus; PNP, polyneuropathy; SNHI, sensorineural hearing impairment.

Additional table 1. List of 562 genes analyzed from exome sequencing data

AAAS	*ATP5C*	*CASK*	*CSTB*	*ERCC5*	*HARS2*	*MAG*
AARS2	*ATP5D*	*CAV1*	*CTBP1*	*ERCC6*	*HEPACAM*	*MAN2B*
ABCB7	*ATP5E*	*CC2D2A*	*CTC1*	*ERLIN1*	*HERC1*	*MAPK10*
ABCD1	*ATP5F1*	*CCDC88C*	*CTDP1*	*ERLIN2*	*HEXA*	*MARS2*
ABHD12	*ATP5G1*	*CDKL5*	*CTSA*	*ETHE1*	*HEXB*	*ADSSL1*
ACO2	*ATP5G2*	*ADA2*	*CTSD*	*EXOSC3*	*HIBCH*	*MECP2*
COQ8A	*ATP5G3*	*CEP41*	*CTSF*	*FA2H*	*HLCS*	*MECR*
ADSL	*ATP5H*	*CEP104*	*CUL4B*	*FARS2*	*HNRNPH2*	*MFSD8*
AFG3L2	*ATPIF1*	*CEP120*	*CWF19L1*	*FASTKD2*	*HSD17B4*	*MGME1*
AHI1	*ATP5I*	*CEP290*	*CYC1*	*FBXL4*	*HSPD1*	*MKKS*
ALDH18A1	*ATP5J*	*CHAMP1*	*CYP2U1*	*FDXR*	*HTRA1*	*MKS1*
ALDH5A1	*ATP5J2*	*CHCHD10*	*CYP27A1*	*FGF12*	*IBA57*	*MLC1*
ALG6	*ATP5MPL*	*CHMP1A*	*CYP7B1*	*FGF14*	*IFT140*	*MMADHC*
ALS2	*ATP5O*	*CLCN2*	*DARS2*	*FKRP*	*INPP5E*	*MME*
AMACR	*ATP7B*	*CLN5*	*DBT*	*FKTN*	*ISPD*	*MPDU1*
ANO10	*ATP8A2*	*CLN6*	*DCX*	*FLVCR1*	*ITM2B*	*MPV17*
AP1S2	*ATPAF2*	*CLN8*	*DDHD1*	*FMR1*	*ITPR1*	*MPZ*
AP4B1	*AUH*	*CLPB*	*DDHD2*	*FOLR1*	*KCNA1*	*MRE11*
AP4E1	*B4GALNT1*	*CLPP*	*DGAT2*	*FOXG1*	*KCNA2*	*MSTO1*
AP4M1	*B9D1*	*COA5*	*DKC1*	*FOXRED1*	*KCNC1*	*MTFMT*
AP4S1	*BBS1*	*COASY*	*DLAT*	*FTL*	*KCNC3*	*MTPAP*
AP5Z1	*BBS2*	*COG4*	*DLD*	*FXN*	*KCND3*	*MTTP*
NAXE	*BBS4*	*COL18A1*	*DMXL2*	*GABRB3*	*KCNJ10*	*MVK*
APOB	*BBS5*	*COQ2*	*DNAJC19*	*GALC*	*KCNQ2*	*NDUFA10*
APOPT1	*BBS7*	*COQ4*	*DNAJC3*	*GAMT*	*KCTD7*	*NDUFA11*
APTX	*BBS9*	*COQ6*	*DNAJC5*	*GAN*	*WASHC5*	*NDUFA12*
ARL6	*BBS10*	*COQ9*	*DNM2*	*GBA2*	*KIAA0556*	*NDUFA2*
ARL13B	*BBS12*	*COX10*	*DNMT1*	*GBE1*	*KIAA0586*	*NDUFA9*
ARL6IP1	*BCKDHA*	*COX15*	*DOCK3*	*GCH1*	*KIDINS220*	*NDUFAB1*
ARMC9	*BCKDHB*	*COX20*	*DPM1*	*GCLC*	*KIF1A*	*NDUFAF1*
ARSA	*BCS1L*	*COX4I1*	*DYNC1H1*	*GFAP*	*KIF1B*	*NDUFAF2*
ARV1	*BEAN1*	*COX4I2*	*EBF3*	*GJA1*	*KIF1C*	*NDUFAF4*
ARX	*BRAT1*	*COX5A*	*EEF2*	*GJB1*	*KIF5A*	*NDUFAF5*
ASL	*BSCL2*	*COX5B*	*EIF2B1*	*GJC2*	*KIF7*	*NDUFAF6*
ASS1	*BTD*	*COX6A2*	*EIF2B2*	*GLB1*	*KY*	*NDUFB1*
ATCAY	*C5ORF42*	*COX6B1*	*EIF2B3*	*GMPPB*	*L1CAM*	*NDUFB2*
ATG5	*TWNK*	*COX6B2*	*EIF2B4*	*GOSR2*	*L2HGDH*	*NDUFB3*
ATL1	*C12orf65*	*COX6C*	*EIF2B5*	*GPAA1*	*LAMA1*	*NDUFB4*
ATM	*C19orf12*	*COX7A1*	*ELOVL4*	*GPI*	*LARS2*	*NDUFB6*
ATM	*C5orf42*	*COX7A2*	*ELOVL5*	*ADGRG1*	*LARGE*	*NDUFB7*
ATP1A2	*CA8*	*COX7B2*	*ENTPD1*	*GRID2*	*LIG4*	*NDUFB8*
ATP1A3	*CACNA1A*	*COX7C*	*EOMES*	*GRM1*	*LMNB1*	*NDUFB9*
ATP13A2	*CACNA1G*	*CP*	*EPM2A*	*GRN*	*LMNB2*	*NDUFB10*
ATP2B3	*CACNB4*	*CPS1*	*SELENOI*	*GSS*	*LRP4*	*NDUFB11*
ATP5A	*CAMTA1*	*CPT1C*	*ERCC3*	*HACE1*	*LRPPRC*	*NDUFC1*
ATP5B	*CAPN1*	*CSPP1*	*ERCC4*	*HARS*	*LRSAM1*	*NDUFC2*

Additional table 1 continues.

NDUFS1	*PGM3*	*RNASEH1*	*SLC6A1*	*TMEM216*	*WWOX*
NDUFS2	*PHYH*	*RNASET2*	*SLC6A19*	*TMEM231*	*XPA*
NDUFS3	*PIBF1*	*RNF168*	*SLC9A1*	*TMEM237*	*XRCC1*
NDUFS4	*PIEZO2*	*RNF170*	*SLC9A6*	*TMEM240*	*XRCC4*
NDUFS6	*PIGG*	*RNF216*	*SNAP25*	*TPK1*	*YME1L1*
NDUFS7	*PIK3R5*	*ROGDI*	*SNX14*	*TPP1*	*ZFYVE26*
NDUFS8	*PLA2G6*	*RPGRIP1L*	*SOD1*	*TRAPPC11*	*ZIC1*
NDUFV1	*PLP1*	*RRM2B*	*SOX10*	*TRIM32*	*ZIC4*
NDUFV2	*PMM2*	*RTN2*	*SPAST*	*TRNT1*	*ZNF423*
NDUFV3	*PMP22*	*RTN4IP1*	*SPG11*	*TRPC3*	*ZNF592*
NF2	*PMPCA*	*RUBCN*	*SPG20*	*TSEN2*	
NHLRC1	*PNKD*	*SACS*	*SPG21*	*TSEN34*	
NIPA1	*PNKP*	*SAMD9L*	*SPG7*	*TSEN54*	
NKX2-1	*PNP*	*SARS*	*SPR*	*TSFM*	
NKX6-2	*PNPLA6*	*SCARB2*	*SPTAN1*	*TTBK2*	
NOL3	*PNPLA6*	*SCN1A*	*SPTBN2*	*TTC8*	
NPC1	*POLG*	*SCN2A*	*SQSTM1*	*TTC19*	
NPC2	*POLR1A*	*SCN8A*	*STUB1*	*TTC21B*	
NPHP1	*POLR1C*	*SCO1*	*STXBP1*	*TTPA*	
NT5C2	*POLR3A*	*SCYL1*	*SUCLG1*	*TTR*	
NUBPL	*POLR3B*	*SDHA*	*SUFU*	*TUBA1A*	
NUP62	*POMGNT1*	*SDHAF1*	*SUMF1*	*TUBB*	
OFD1	*POMGNT2*	*SDHB*	*SUOX*	*TUBB4A*	
OPA1	*POMT1*	*SDHC*	*SURF1*	*TYMP*	
OPA3	*POMT2*	*SDHD*	*SYNE1*	*UCHL1*	
OPHN1	*PPP2R2B*	*SEPSECS*	*SYT14*	*UQCR10*	
OTC	*PPT1*	*SERAC1*	*TACO1*	*UQCR11*	
OTUD4	*PRDM8*	*SETX*	*TBC1D23*	*UQCRB*	
PANK2	*PRF1*	*SH3TC2*	*TBC1D24*	*UQCRC1*	
PAX6	*PRICKLE1*	*SIL1*	*TBCE*	*UQCRC2*	
PC	*PRICKLE2*	*SLC1A3*	*TCF4*	*UQCRFS1*	
PCDH19	*PRKCG*	*SLC13A5*	*TCTN1*	*UQCRH*	
PDE6D	*PRNP*	*SLC16A2*	*TCTN2*	*UQCRQ*	
PDHA1	*PRPS1*	*SLC17A5*	*TCTN3*	*UROC1*	
PDHB	*PRRT2*	*SLC19A2*	*TDP1*	*VAMP1*	
PDSS1	*PRX*	*SLC19A3*	*TDP2*	*VARS2*	
PDSS2	*PSAP*	*SLC2A1*	*TECPR2*	*VLDLR*	
PDYN	*PSEN1*	*SLC9A6*	*TELO2*	*VRK1*	
PET100	*PTRH2*	*SLC20A2*	*TFG*	*VWA3B*	
PEX1	*PTS*	*SLC25A46*	*TGM6*	*WARS2*	
PEX10	*PUM1*	*SLC30A9*	*TH*	*WDPCP*	
PEX16	*RARS*	*SLC33A1*	*THG1L*	*WDR26*	
PEX2	*RARS2*	*SLC39A4*	*TINF2*	*WDR62*	
PEX26	*REEP1*	*SLC46A1*	*TMEM67*	*WDR73*	
PEX7	*RELN*	*SLC52A2*	*TMEM70*	*WDR81*	
PGK1	*RFT1*	*SLC52A3*	*TMEM107*	*WFS1*	
